# Energy, amino acid, and phosphorus digestibility and energy prediction of thermally processed food waste sources for swine^1^

**DOI:** 10.1093/tas/txz028

**Published:** 2019-04-05

**Authors:** Leonard Fung, Pedro E Urriola, Gerald C Shurson

**Affiliations:** Department of Animal Science, University of Minnesota, St. Paul, MN

**Keywords:** amino acids, digestibility, energy, food waste, phosphorus, swine

## Abstract

Recycling energy and nutrients from food waste into animal feed decreases the environmental impact of food animal production. However, recycling energy and nutrients from various food waste sources into swine feeding programs is constrained by the high variability and lack of data on the digestibility of energy and nutrients. Therefore, the objectives of this study were to evaluate the digestibility of energy, amino acids, and phosphorus in thermally dried food waste sources fed to growing pigs and to compare in vivo determined digestibility values with those obtained from in vitro digestibility procedures and published prediction equations to determine the accuracy of using these nutritional evaluation methods. Pigs (*n* = 36; initial body weight = 16.37 ± 1.9 kg) were utilized to determine digestible energy (DE) and metabolizable energy (ME) content, as well as standardized total tract digestibility (STTD) of phosphorus and standardized ileal digestibility (SID) of amino acids in three sources of dehydrated food waste in three separate trials. Initial body weight of pigs at the beginning of each digestibility trial was used as the blocking factor in a randomized complete block design. Diets were formulated to contain 30% food waste derived from fish waste (FW), supermarket waste (containing bakery, fruits and vegetables, meat, and deli foods from a single supermarket; SMW), and fruit and vegetable waste (FVW). The DE and ME content of FW (DE = 5,057 kcal/kg; ME = 4,820 kcal/kg) and SMW (DE = 5,071 kcal/kg; ME = 4,922 kcal/kg) were not different (*P* > 0.05), whereas FVW had the least (*P <* 0.05) DE (2,570 kcal/kg) and ME (2,460 kcal/kg) content compared with FW and SMW. Digestibility of crude protein and amino acids was greater (*P <* 0.05) in FW and SMW compared with FVW. The in vitro digestibility procedure can be used to approximate the digestibility of dry matter (DM) and energy in SMW, FW, and FVW compared with in vivo estimates, but significant error exists depending on the chemical characteristics of each food waste source. However, use of the prediction equations and digestibility data obtained from the in vitro procedure resulted in high accuracy in estimating DE content of FW (observed = 5,058 kcal/kg DM vs. predicted = 4,948 kcal/kg DM), SMW (observed = 5,071 kcal/kg DM vs. predicted 4,978 kcal/kg DM), and FVW (observed = 2,570 kcal/kg DM vs. predicted 2,814 kcal/kg DM) sources.

## INTRODUCTION

In the United States, food waste accounts for 21.6% of the discarded municipal solid waste, and only 5% of food waste generated is diverted away from landfills annually (U.S. Environmental Protection Agency, 2014). As a result, there is increasing interest in utilizing food waste as animal feed because of its environmental benefits, low cost, and diversion from low-value landfill disposal to higher-value animal feed products (Esteban et al., 2007; Salemdeeb et al., 2017).

Feed cost accounts for about 65% to 75% of the total cost of pork production (Thaler and Holden, 2010). Increased use and prices of grain and lipids in biofuel production have contributed to increased interest in using lower cost alternative feed ingredients in commercial swine diets (Woyengo et al., 2014). In the United States, most commercial pork production systems use dry feeding rather than liquid feeding systems, and diets are based on grains, soybean meal, and various byproducts (e.g., distillers dried grains with solubles, wheat middlings, bakery waste; Richert and DeRouchey, 2010). Limited studies have evaluated the nutritional value of feeding wet (Jinno et al., 2018) or dried post-consumer food waste to growing pigs (Westendorf et al., 1998; Myer et al., 1999). However, results from these previous studies suggest that inclusion of dried food waste in practical swine diets had little to no effect on growth performance and carcass composition of pigs compared with feeding standard corn-soybean-based diets (Westendorf et al., 1998). However, these studies evaluated only one food waste source, which was not representative of the wide variety of food waste sources produced in various segments of the food chain. Therefore, more food waste sources with varying nutritional characteristics need to be evaluated for their potential use in swine feeding programs.

To meet the daily energy and digestible nutrient requirements of pigs, information on the digestible energy (DE) and metabolizable energy (ME) content, standardized ileal digestibility (SID) of amino acids (AA), and standardized total tract digestibility (STTD) of phosphorus is needed for all feed ingredients being fed (NRC, 2012). There are no published in vivo data for DE, ME, SID of AA, or STTD of phosphorus for various food waste sources. Likewise, there are no data on the accuracy of estimating DE, ME, SID of AA, or STTD of phosphorus using in vitro assays, or predicting DE and ME content from published equations. Therefore, the objective of the study was to determine the concentration of DE and ME, as well as SID of AA and STTD of phosphorus of three sources of thermally processed food waste and to compare in vivo determined values with those derived from in vitro digestibility determinations as well as prediction equations based on chemical composition of the food waste sources for swine.

## MATERIALS AND METHODS

The Institutional Animal Care and Use Committee at the University of Minnesota reviewed and approved protocol #1601-34068A for these experiments.

### Dehydrated Food Waste Sources and Chemical Analysis

Three dehydrated food waste sources (fish waste [FW]; supermarket waste [SMW]; and fruit and vegetable waste [FVW]) were collected and processed by TUBS, Inc. (Minneapolis, MN) for use in this study. The FW was obtained from a single fish processing facility in Minnesota, and the FVW was collected from a local fruit and vegetable processing plant. The SMW was composed of a mixture of fruits and vegetables, deli foods, meat, and bakery products from a local supermarket. Three composite samples were collected at the supermarket over a 3-wk period, and each collection consisted of waste collected over a 2-d period from the four departments and stored in 120-L barrel. Thus, the final product was a mixture of food waste representing a total of 6 d from the four departments. After collecting the raw materials from their respective sources, the three food waste sources were ground and mixed individually through an auger screw press and dehydrated using a drum dryer to achieve a final moisture content of less than 20%. Samples were then stored in 20-L buckets at −4 °C before submitting for chemical analysis.

The three dehydrated sources of food waste were subsampled and submitted to the University of Missouri Agricultural Experiment Station Chemical Laboratories (Columbia, MO) for chemical analyses (Table 1). Samples were analyzed using AOAC (2012) procedures for AA profile (Method 982.30), acid detergent fiber (ADF; Method 973.18), crude protein (CP; Method 984.13), ether extract (EE; Method 920.39), ash (Method 942.05), dry matter (DM; Method 934.01), phosphorus (Method 966.01), and calcium (Ca; Method 980.02). Neutral detergent fiber (NDF) was analyzed as described by Van Soest (1991), and thiobarbituric reactive substances (TBARS) as described by Wrolstad (2001). TBARS were measured in all food waste samples because of the potential for lipid peroxidation before processing, as well as during the heating and dehydration processes. The in vivo determinations of DE, ME, SID AA, and STTD P were conducted in three separate experiments, and the same batch of each source of food waste was used in all experiments. Pigs were weighed between experiments to calculate the daily feed allowance based on initial body weight (BW).

**Table 1.** Analyzed gross energy and nutrient composition of fish waste (FW), supermarket waste (SMW), fruits and vegetable waste (FVW), and corn (as-fed basis)

	Ingredient			
Item	FW	SMW	FVW	Corn
Dry matter, %	92.16	82.89	90.50	85.94
Gross energy, kcal/kg	5,876	5,235	3,731	3,943
Crude protein, %	57.59	24.39	9.17	6.88
Crude fat, %	17.38	29.05	1.29	2.39
Ash, %	15.05	3.47	5.06	1.07
Acid detergent fiber, %	3.40	16.47	20.82	3.09
Neutral detergent fiber, %	3.81	18.50	28.20	7.85
Ca, %	4.83	0.28	0.38	0.01
P, %	2.72	0.31	0.24	0.27
Indispensable AA^1^, %				
Arg	3.62	1.19	0.35	0.28
His	1.32	0.60	0.14	0.19
Ile	2.21	1.14	0.31	0.26
Leu	3.59	1.79	0.44	0.84
Lys	3.79	0.68	0.34	0.23
Met	1.45	0.37	0.10	0.10
Phe	2.06	0.99	0.32	0.35
Thr	2.22	0.92	0.25	0.22
Trp	0.57	0.12	0.04	0.05
Val	2.59	1.19	0.36	0.32
Total	23.41	9.00	2.65	2.85
Dispensable AA, %				
Ala	3.94	1.35	0.40	0.51
Asp	4.86	2.01	0.81	0.46
Cys	0.41	0.27	0.11	0.14
Glu	7.11	3.91	0.99	1.24
Gly	5.74	1.42	0.36	0.27
Pro	3.23	1.34	0.42	0.60
Ser	2.00	0.75	0.25	0.29
Tyr	1.78	0.78	0.17	0.16
Total	29.08	11.82	3.51	3.69

^1^AA = amino acid.

### Energy Balance and Concentration of DE and ME

#### Diets, animals, and experimental design.

The first experiment was designed to determine the DE and ME content in FW, FVW, and SMW. Thirty-six growing barrows (initial BW = 16.37 ± 1.9 kg) were housed individually in metabolism crates equipped with a stainless-steel feeder and nipple waterer, using a randomized complete block design with initial BW as the blocking factor. Pigs within block were assigned randomly to one of four dietary treatments consisting of a basal control diet containing 96.9% corn and three test diets consisting of 30% of each respective food waste source to replace corn in the basal diet (Table 2). Titanium dioxide was added at 0.40% to each diet to serve as an indigestible marker for use in digestibility calculations. Vitamins and minerals were included in the diets to meet or exceed requirements for growing pigs based on 15 kg BW (NRC, 2012).

**Table 2.** Diet composition and analyzed gross energy and chemical content of experimental diets containing fish waste (FW), supermarket waste (SMW), fruit and vegetable waste (FVW), and corn used in the energy balance experiment (as-fed basis)

Item	FW	SMW	FVW	Control
Ingredient, %				
Corn	66.90	66.90	66.90	96.90
Food waste source	30.00	30.00	30.00	0.00
Dicalcium phosphate	1.15	1.15	1.15	1.15
Limestone	0.85	0.85	0.85	0.85
Salt	0.40	0.40	0.40	0.40
VTM premix^1^	0.30	0.30	0.30	0.30
Titanium dioxide	0.40	0.40	0.40	0.40
Total	100.00	100.00	100.00	100.00
Analyzed composition				
Dry matter, %	87.73	86.76	87.67	86.53
Gross energy, kcal/kg	4,172	4,108	3,786	3,821
Crude protein, %	15.97	10.12	7.11	5.27
Ether extract, %	12.65	11.10	12.13	2.54
Titanium, %	0.25	0.22	0.23	0.21

^1^VTM = vitamin trace mineral. The premix provided the following per kilogram of complete diet: vitamin A, 12,000 IU; vitamin D3, 2,500 IU; vitamin E, 30 IU; vitamin K3, 3 mg; vitamin B12, 0.012 mg; riboflavin, 4 mg; niacin, 40 mg; pantothenic acid, 15 mg; choline chloride, 400 mg; folic acid, 0.7 mg; thiamin, 1.5 mg; pyridoxine, 3 mg; biotin, 0.1 mg; Zn, 105 mg; Mn, 22 mg; Fe, 84 mg; Cu, 10 mg; I, 0.50 mg; Se, 0.35 mg.

#### Feeding and sample collection.

Pigs were fed the experimental diets for 9 d, which included a 5-d adaptation period followed by a 4-d feces and urine collection period. Daily feed allowance was calculated according to three times the maintenance energy requirement of the smallest pig in each treatment (197 kcal ME/kg of BW^0.60^; NRC, 2012) and was divided and fed in two equal meals at 0800 and 1600 h. All pigs had ad libitum access to water from nipple drinkers. Representative samples of feces excreted were collected twice daily starting from 0800 h on day 6 to day 13 and stored immediately at −20 °C after collection until further analyses. Urine collection was initiated at 1600 h on day 5 by placing buckets under the collection pan of each metabolism crate. Urine was collected daily, and 50 mL of 3 N HCl was added to each collection container before each collection day through day 13. The total volume of urine was measured daily, and about 10% of the total volume was subsampled, filtered through glass wool, and stored at −20 °C until further analyses.

#### Chemical analyses.

After the 4-d collection period, fecal samples were dried at 65 °C in a forced-air oven for 24 h and ground through a 2-mm screen. Urine samples were thawed and mixed before subsampling for drying in a forced-air oven at 55 °C for 24 h (Jacobs et al., 2011). Fecal and urine samples were analyzed in duplicates for gross energy (GE) using an isoperibol bomb calorimeter (Parr 6400; Parr Instrument Company, Moline, IL). Diets and fecal subsamples were also submitted to the University of Missouri Agricultural Experiment Station Chemical Laboratories and analyzed for ADF, NDF, CP, EE, ash, and DM as previously described. Diets and fecal samples were also analyzed for titanium dioxide (Myers et al., 2004).

#### Calculations and statistical analysis.

DE and ME content of the diets was determined by the difference method relative to the proportion of indigestible marker content (Adeola, 2001). The individual pig was considered as the experimental unit and data were analyzed using the Mixed procedure of SAS (SAS Inst. Inc., Cary, NC). Dietary treatments were fixed effects and block was considered as a random effect. Data are presented as the least squared means using the Tukey adjustment for multiple comparisons. The univariate procedure of SAS was used to search for outliers and patterns in studentized residuals. Significance was noted when *P ≤* 0.05, and trends were noted at 0.05 ≤ *P* ≤ 0.10.

### Phosphorus Digestibility

#### Diets, animals, and experimental design.

The objective of the second experiment was to determine the apparent total tract digestibility (ATTD) and STTD of phosphorus of the three food waste ingredients. The same 36 growing barrows used in the energy balance experiment (initial BW = 15.87 ± 2.3 kg) were weighed after that experiment and continued to be individually housed in metabolism crates equipped with a stainless-steel feeder and nipple waterer. A randomized complete block design was used, which consisted of three dietary treatments providing 12 replicates per treatment. Individual BW of the pigs was used as the blocking factor. Three diets were formulated to contain 30% of the test ingredients (FW, SMW, and FVW), 49.9% corn starch, and 15% sucrose (Table 3). Food waste ingredients provided the only source of P in the diets. Titanium dioxide was added at 0.40% of the diet as an indigestible marker, which was used to determine P digestibility by difference (Agudelo et al., 2010; Zhang and Adeola, 2017). Vitamins and minerals were included in the diets to meet or exceed the requirements for growing pigs based on 20 kg BW (NRC, 2012).

**Table 3.** Diet composition and analyzed gross energy and nutrient content of diets used in the phosphorus digestibility experiment (as-fed basis)

Item	FW^1^	SMW	FVW
Ingredient, %			
Corn starch	49.90	49.90	49.90
Food waste	30.00	30.00	30.00
Sucrose	15.00	15.00	15.00
Soybean oil	3.00	3.00	3.00
Limestone	1.00	1.00	1.00
Salt	0.40	0.40	0.40
VTM premix^2^	0.30	0.30	0.30
Titanium dioxide	0.40	0.40	0.40
Total	100.00	100.00	100.00
Analyzed composition			
Dry matter, %	92.78	91.58	92.66
Ca, %	1.55	0.63	0.65
P, %	0.57	0.13	0.08
Ash, %	5.12	2.87	3.09
Neutral detergent fiber %	0.74	0.79	7.16
Acid detergent fiber, %	0.68	0.55	5.47
Titanium, %	0.22	0.23	0.20
Gross energy, kcal/kg	4,366	4,051	4,001

^1^FW = fish waste; SMW = supermarket waste; FVW = fruits and vegetable waste.

^2^VTM = vitamin trace mineral. The premix provided the following per kilogram of complete diet: vitamin A, 12,000 IU; vitamin D3, 2,500 IU; vitamin E, 30 IU; vitamin K3, 3 mg; vitamin B12, 0.012 mg; riboflavin, 4 mg; niacin, 40 mg; pantothenic acid, 15 mg; choline chloride, 400 mg; folic acid, 0.7 mg; thiamin, 1.5 mg; pyridoxine, 3 mg; biotin, 0.1 mg; Zn, 105 mg; Mn, 22 mg; Fe, 84 mg; Cu, 10 mg; I, 0.50 mg; Se, 0.35 mg.

#### Feeding and sample collection.

Pigs were fed their assigned experimental diets for 9 d, which included a 5-d adaptation period followed by a 4-d fecal collection period. Daily feed allowance was calculated based on three times the maintenance energy requirement of the smallest pig in each treatment and was equally divided into two equal meals that were fed at 0800 and 1600 h. All pigs had ad libitum access to water. Fecal samples were collected twice daily starting from 0800 h on day 6 and stored immediately at −20 °C after collection.

#### Chemical analyses.

After completing the 4-d total collection period, fecal samples were dried at 65 °C in a forced-air oven for 24 h and ground finely to pass a 2-mm screen. Diets were analyzed for titanium, DM, ash, Ca, P, ADF, NDF and GE as previously described, and fecal samples were analyzed for titanium, DM and P.

#### Calculations and statistical analysis.

The ATTD of P was calculated according to the difference method described by Agudelo et al. (2010), and the STTD was calculated by subtracting a constant basal endogenous loss of P, which was estimated to be 190 mg/kg DM intake (NRC, 2012).

Individual pig was used as the experimental unit, and data were analyzed using the Mixed procedure of SAS with Tukey adjustment for mean separation. Dietary treatments were fixed effects and block was considered as a random effect. Significance was noted when *P* ≤ 0.05, and trends were noted when 0.05 ≤ *P* ≤ 0.10.

### AA Digestibility

#### Diets, animals, and experimental design.

The objective of the third experiment was to determine the apparent ileal digestibility (AID) and SID of AA of the three food waste sources. The 36 growing barrows used in the energy balance and phosphorus digestibility experiments were also used in the AA digestibility experiment. Upon the completion of the phosphorus digestibility experiment, pigs (initial BW = 21 ± 3.5 kg) were surgically fitted with a T-cannula at the distal ileum. Pigs were individually housed in metabolism crates, in a randomized complete block design (blocks were based on initial pig BW) with four dietary treatments to provide nine replicates per treatment. Three corn starch-based diets contained 30% food waste from either FW, SMW, or FVW as the sole source of AA, and one nitrogen-free diet to estimate the basal endogenous losses of CP and AA, were fed. Titanium dioxide was included at 0.40% of each diet as an indigestible marker for AA digestibility calculations as described in Stein et al. (2007). Vitamins and minerals were included in the diets to meet or exceed requirements for growing pigs based on 25 kg BW (NRC, 2012).

#### Feeding and sample collection.

Pigs were fed their assigned experimental diets for 7 d, which included a 5-d adaptation period followed by a 2-d ileal digesta collection period. Daily feed allowance was calculated to be equivalent to three times the maintenance energy requirement of the pig with the lowest BW in each treatment, and was equally divided into two meals fed at 0800 and 1600 h. All pigs had ad libitum access to water. Ileal digesta were collected for 8 h on days 6 and 7, beginning at 0800 h and continuing until 1600 h. A 207-mL bag (Whirl-pack, Nasco, Fort Atkinson, WI) was attached to the barrel of the cannula using a cable zip-tie during total collection of ileal digesta samples. Bags were replaced whenever they were filled or at 30-min intervals. All samples were stored at −20 °C before analysis.

#### Chemical analyses.

After the 2-d collection, digesta samples were thawed, mixed, and subsampled before lyophilization for 5-d, and dried samples were subsequently ground to pass a 2-mm screen. Diets were analyzed for AA profile, titanium, DM, ash, ADF, NDF, and GE content as previously described, and digesta samples were analyzed for AA profile, DM, and titanium concentrations (Table 4).

**Table 4.** Diet composition and analyzed gross energy, nutrient, and amino acid (AA) content of diets used in the AA digestibility experiment (as-fed basis)

Item	FW^1^	SMW	FVW	N free
Ingredient, %				
Corn starch	43.95	43.95	43.95	67.80
Food waste	30.00	30.00	30.00	0.00
Sucrose	20.00	20.00	20.00	20.00
Soybean oil	3.00	3.00	3.00	4.00
Dicalcium phosphate	1.10	1.10	1.10	2.15
Limestone	0.85	0.85	0.85	0.45
Titanium dioxide	0.40	0.40	0.40	0.40
Salt	0.40	0.40	0.40	0.40
VTM premix^2^	0.30	0.30	0.30	0.30
Potassium carbonate	—	—	—	0.40
Magnesium oxide	—	—	—	0.10
Solka-Floc^3^	—	—	—	4.00
Analyzed composition				
Dry matter, %	92.67	92.38	93.16	93.17
Crude protein, %	13.19	6.12	2.84	0.38
Neutral detergent fiber %	1.30	0.79	9.03	1.61
Acid detergent fiber, %	0.19	0.49	5.78	1.35
Gross energy, kcal/kg	3,990	3,991	3,569	3,375
Indispensable AA, %				
Arg	0.80	0.30	0.09	0.01
His	0.31	0.17	0.04	0.00
Ile	0.51	0.27	0.08	0.01
Leu	0.84	0.44	0.15	0.04
Lys	0.93	0.40	0.10	0.02
Met	0.33	0.11	0.03	0.01
Phe	0.48	0.23	0.09	0.02
Thr	0.52	0.22	0.07	0.01
Trp	0.12	0.07	0.03	0.02
Val	0.57	0.29	0.10	0.01
Total	4.58	2.17	0.72	0.13
Dispensable AA, %				
Ala	0.92	0.33	0.12	0.02
Asp	1.12	0.50	0.24	0.02
Cys	0.09	0.06	0.03	0.01
Glu	1.62	0.99	0.33	0.05
Gly	1.35	0.33	0.10	0.02
Pro	0.83	0.34	0.14	0.03
Ser	0.47	0.21	0.08	0.01
Tyr	0.34	0.16	0.04	0.01
Total	6.76	2.93	1.08	0.16

^1^FW = fish waste; SMW = supermarket waste; FVW = fruits and vegetable waste.

^2^VTM = vitamin trace mineral. The premix provided the following per kilogram of complete diet: vitamin A, 12,000 IU; vitamin D3, 2,500 IU; vitamin E, 30 IU; vitamin K3, 3 mg; vitamin B12, 0.012 mg; riboflavin, 4 mg; niacin, 40 mg; pantothenic acid, 15 mg; choline chloride, 400 mg; folic acid, 0.7 mg; thiamin, 1.5 mg; pyridoxine, 3 mg; biotin, 0.1 mg; Zn, 105 mg; Mn, 22 mg; Fe, 84 mg; Cu, 10 mg; I, 0.50 mg; Se, 0.35 mg.

^3^International Fiber Corporation, North Tonawanda, NY.

#### Calculations and statistical analysis.

Endogenous losses of CP and AA, as well as AID and SID of the food waste ingredients were calculated as described by Stein et al. (2007) using an indigestible marker. Individual pig was used as the experimental unit, and data were analyzed by the Mixed procedure of SAS using model and analysis described for experiment 1.

### In Vitro DM and Energy Digestibility of FW, SMW, FVW, and corn

Samples of FW, SMW, FVW, and corn were analyzed using a three-step in vitro enzymatic hydrolysis and fermentation procedure to determine the in vitro digestibility of DM and energy (Huang et al., 2017). In vitro data obtained from these analyses were compared with in vivo data to determine the applicability of using the in vitro procedure to predict the feeding values of FW, SMW, and FVW.

#### In vitro enzymatic hydrolysis.

In vitro enzymatic hydrolysis was performed to simulate the conditions of apparent ileal digestion of FW, SMW, FVW, and corn. Samples of FW, SMW, FVW, and corn were ground using a mortar and pestle to reduce particle size before subjecting them to in vitro enzymatic hydrolysis using pepsin and pancreatin according to the procedure of Boisen and Fernandez (1997). After grinding, 2 g of each sample (*n* = 8) was transferred into 500-mL conical flasks with a phosphate buffer solution (100 mL, 0.1 M, pH 6.0), and HCl solution (40 mL, 0.2 M) was added. The pH of the solution was adjusted to 2.0 using 1 M HCl or 1 M NaOH, and 2 mL of a chloramphenicol (Sigma C-0378, Sheboygan Falls, WI) solution (0.5 g 100 mL/L ethanol) was added to inhibit microbial activity. Fresh porcine pepsin solution (4 mL, 25 g/L, Sigma P-7000, Sheboygan Falls, WI) was subsequently added to the flasks with rubber stoppers and placed in a 39 °C water bath for 2 h. After pepsin hydrolysis, 40 mL of phosphate buffer (0.2 M, pH 6.8) and 20 mL of 0.6 M NaOH were added to the flasks, and the pH of the solution was adjusted to 6.8 using 1 M HCl or 1 M NaOH. Fresh pancreatin solution (2 mL, 100 g/L pancreatin, Sigma P-1750, St. Louis, MO) was then added, and the flasks were placed in a 39 °C water bath for 4 h. After the hydrolysis period was complete, residues were collected by filtration using a nylon bag (42 µm; Ankom Technologies, Macedon, NY) and washed with ethanol (2 × 25 mL 95% ethanol) and acetone (2 × 25 mL 99.5% acetone). Residues in the bags were then dried in forced-air oven at 60 °C for 48 h and subsequently weighed. Hydrolyzed residues from the same treatments (*n* = 4) were pooled for subsequent in vitro fermentation. The remaining four replicates were stored individually for GE determination using an isoperibol bomb calorimeter (model 1281; Parr Instrument Co., Moline, IL).

#### In vitro fermentation.

In vitro fermentation was conducted to simulate the in vivo fermentation of FW, SMW, FVW, and corn in the hindgut of pigs. The residues of each sample after hydrolysis were used as substrates. Rate of fermentation was monitored using a cumulative gas production technique by Bindelle et al. (2007). Two hundred milligram of hydrolyzed residue from each treatment (*n* = 4) was transferred into 125-mL glass bottles and inoculated with 30-mL buffer solution containing macro- and mirco-minerals (Menke and Steingass, 1988) and fecal inoculum. Feces were obtained from pigs (BW = 120 ± 2 kg) from the Cargill Innovation Campus (Elk River, MN) that were fed a corn, wheat middlings, and soybean meal diet without antibiotics. Feces were collected through rectal stimulation, and samples were placed immediately into an air tight bag that was then stored at 39 °C for 1 h until inoculum preparation was completed using 0.05 g of feces/mL of buffer solution. Fecal inocula were then filtered through a 250-µm screen and transferred into the bottles containing hydrolyzed residues. Fermentation bottles were sealed with rubber stoppers and placed in water bath at 39 °C for incubation. An anaerobic environment was maintained throughout the incubation period by adding CO_2_ gas. Gas production was measured at 0, 2, 5, 8, 12, 18, 24, 36, 48, and 72 h to monitor the rate of fermentation. The bottles were vented after each measurement, and at the end of 72 h, supernatant from each bottle was collected and frozen before analysis for volatile fatty acids (VFA).

#### Chemical analysis.

GE of the hydrolyzed residue was determined using an adiabatic bomb calorimeter (Parr 6400; Parr Instrument Company, Moline, IL) with benzoic acid used as standard. VFA concentrations of the supernatant collected from the fermentation procedure were measured using gas chromatography (Agilent 6890 system, Germany). Two milliliters of supernatant from each bottle (*n* = 4) collected from the 72-h fermentation period was transferred into 10-mL centrifuge tubes and mixed with 2-mL 50% sulfuric acid, 0.4-g sodium chloride, 0.4-mL internal standard, and 2-mL diethyl ether. The mixtures were then vortexed for 2 min and centrifuged at 3,000 × *g* for 5 min. Finally, the supernatant of the etheric layer was transferred into autosampler vials before loading into the gas chromatograph–mass spectrometer (Agilent 6890 system) for VFA analysis.

#### Calculations and statistical analysis.

In vitro DM digestibility (IVDMD) was calculated as follows:

$$\text{IVDMD }=\;\frac{\left(\text{dry weight of sample before hydrolysis or fermentation - dry weight of the residue after hydrolysis or fermentation}\right)}{\text{dry weight of the sample before hydrolysis or fermentation}}\;$$

Total tract DM digestibility was calculated as follows:

(100 − IVHDMD)×IVFDMD+IVHDMD

where IVHDM denotes in vitro hydrolysi*s* DM digestibility and IVFDM denotes in vitro fermentation DM digestibility expressed as a percentage.

In vitro total tract DE was calculated as the sum of the calculated DE from the hydrolysis procedure and energy released from VFA during fermentation as follows:

$$\begin{array}{cc} \text{In vitro total tract DE} & =\text{ GE of sample before hydrolysis - GE of hydrolysis residue }\\ & +\text{ VFA energy release from fermentation residue} \end{array}$$

Energy released from VFA (acetic, propionic, butyric, and valeric acids) was assumed to be 0.209, 0.365, 0.522, and 0.678 Mcal/mol, respectively (Weast, 1997). Concentrations of VFAs in the supernatent obtained after the fermentation procedure were multiplied by the assumed energy release values to obtain the total energy release from VFAs. The total tract in vivo DE was then calculated as the sum of the energy values obtained from the hydrolysis and VFA production from fermentation. Data were analyzed by the GLM Procedure of SAS, with experiment methods (in vivo or in vitro) and food waste sources considered as fixed effects. Significance was noted when *P* ≤ 0.05, and trends were noted at 0.05 ≤ *P* ≤ 0.10.

### Evaluation of DE and ME Prediction Equations

The applicability of currently available prediction equations for DE and ME was evaluated to determine whether these equations provide an accurate, fast, and less expensive method to estimate the DE and ME content of FW, SMW, and FVW for swine. Energy prediction equations from Noblet and Perez (1993), and stepwise regression equations for DE and ME from Kerr et al. (2017), were evaluated for their accuracy and precision in estimating the DE and ME content of FW, SMW, and FVW based on their chemical composition. GE (kcal/kg DM) was estimated according to the chemical composition of the ingredient (Ewan, 1989):

GE=4,143+(56×%EE)+(15×%CP)−(44×% ash)

The concentrations of DE and ME (kcal/kg DM) of food waste sources were calculated using the following equations from Noblet and Perez (1993), where all input variables are expressed as g/kg DM, and GE, DE, and ME are expressed as kcal/kg DM:

DE=1,161+(0.749×analyzed GE)−(4.3×ash)−(4.1×NDF) (1)

DE=1,161+(0.749×calculated GE)−(4.3×ash)−(4.1×NDF) (2)

DE=4,168−(9.1×ash)+(1.9 × CP)+(3.9×EE)−(3.6×NDF) (3)

ME=4,194−(9.2×ash)+(1.0 × CP)+(4.1 × EE)−(3.5 × NDF) (4)

ME=(1.00 × DE  ​​[​​ 1 ​​]​​ )−(0.68 × CP) (5)

ME=(1.00 × DE [2])−(0.68×CP) (6)

ME=(1.00 × DE [3])−(0.68 × CP) (7)

Stepwise regression equations for calculating DE (equations 1 to 4) and ME (equation 9 to 12) from Kerr et al. (2017) were also used, where all input variables are expressed as % (DM basis), and GE, DE, and ME are expressed as kcal/kg DM as follows:

DE=(GE×1.26)−2,468 (8)

DE=(CP × 56.1)+(EE × 73.4)+(ash × −12.5)−669 (9)

DE=(ash×−87.5)+5,420 (10)

DE=(CP×46.7)+(EE×59.2)+(ash×−36.5)+665 (11)

ME= (GE×1.15)−2,331 (12)

ME=(CP×48.1)+(EE×75.9)+(ash×−18.0)−443 (13)

ME=(ash×−84.0)+4,996 (14)

ME=(CP×36.8)+(EE×49.7)+(ash×−43.1)+1,192 (15)

Chemical composition of FW, SMW, and FVW was used as input variables for the equations, and the calculated values were used to compare with the observed in vivo DE and ME content determined in the energy balance experiments (Tables 8 and 9).

In addition, equations that included in vitro organic matter (OM) digestibility and selected chemical composition inputs from Noblet and Jaguelin-Peyraud (2007) were also used to compare predicted DE (MJ/kg DM) vs. in vivo determined DE content of food waste sources using the following equations (Table 10):

DE=0.0189 OMdv (16)

DE=1.12+0.0168 OMdv+0.0184 EE (17)

DE=5.02+0.0127 OMdv+0.0172 EE − 0.0124 CF (18)

DE=6.05+0.0116 OMdv+0.0166 EE − 0.0135 ADF (19)

where all inputs are expressed as g/kg DM, and OMdv denotes in vitro digestibility of OM (g/kg DM). Calculated DE values from these equations were converted from MJ/kg DM to kcal/kg DM for comparison purposes by using the conversion factor of 1 MJ = 238.834 kcal (U.S. Energy Information Administration, 2017).

### Statistical Analysis

DE and ME values obtained from the prediction equations were compared with the observed values from the in vivo energy balance experiment with a defined range of 95% confidence interval of the observed population. Accuracy was determined by whether the predicted values from the equations fall within the upper and lower boundaries of the calculated margin of error, based on a 95% confidence interval from the values obtained from the in vivo energy digestibility experiment.

## RESULTS AND DISCUSSION

### Chemical Composition of Food Waste

The concentration of GE in FW and SMW was greater than in FVW, which was likely due to the greater concentration of CP and EE in both the FW and SMW compared with FVW (Table 1). The FW contained the greatest concentration of CP because it consisted only of fish carcass remains that contain a substantial amount of protein (Wilson and Cowey, 1985). In fact, the CP content in FW (57.6%) was similar to fish meal (67.5%) reported in NRC (2012). The lipid (EE) content was greatest in SMW because of the relatively high oil content in deli waste and fat trimmings from the meat department of the supermarket. The concentration of minerals was also greater in the FW compared with SMW and FVW. The greater total mineral (ash), Ca, and P content in FW was mainly due to the large proportion of bones and scales in the FW source (Martínez-Valverde et al., 2000) and was comparable to the concentrations in fish meal reported by NRC (2012). FW also had greater concentrations of Lys, Trp, and Met compared with SMW and FVW. However, FW had slightly less Lys, Trp, and Met than the concentrations in fish meal reported in NRC (2012). Thus, the energy and nutrient concentration of the FW source evaluated in this study was similar to that of commercial fish meal currently used in swine nursery diets. The SMW was a mixture of different types of food materials including meat, vegetables, bakery goods, and cooked foods, which resulted in a greater CP content than in FVW, but less than FW. As expected, the FVW source had the least energy, CP, EE, and mineral content because fruits and vegetables are known to contain relatively low amounts of these nutrients and a greater concentration of fiber compared with fish and meat (Greenfield and Southgate, 2003).

### In Vivo DE and ME Content of Food Waste Sources

The concentrations of DE and ME in FW and SMW were greater (*P <* 0.01) than in corn (Table 5), but there were no differences between FW and SMW. In fact, the ME content of FW (4,820 kcal/kg DM) was greater than the NRC (2012) value for fish meal (3,765 kcal/kg DM), and the ME content of FW and SMW (4,922 kcal/kg) was greater than the ME content of full-fat soybeans (4,264 kcal/kg DM) and bakery meal (4,247 kcal/kg DM) reported by NRC (2012). However, although the DE (3,928 kcal/kg DM) and ME (3,875 kcal/kg DM) concentrations in corn were greater (*P <* 0.01) than in FVW (DE = 2,570 kcal/kg DM; ME = 2,460 kcal/kg DM), this source of FVW had greater ME content than soybean hulls (2,139 kcal/kg DM) as reported by NRC (2012). The concentrations of DE and ME in corn obtained in this experiment were similar to those reported in other studies (NRC, 2012; Rojas and Stein, 2013; Oliveira and Stein, 2016). The relatively high DE and ME content in FW and SMW were likely due to the greater concentration of CP and EE in these two food waste sources compared with corn and FVW. In contrast, the low concentrations of DE and ME in FVW are probably a result of the greater concentrations of NDF and ADF, which reduce the digestibility of energy in feed ingredients (Noblet and Le Goff, 2001; Wenk, 2001; Le Gall et al., 2009). These results suggest that both FW and SMW can be used as excellent energy sources in swine diets.

**Table 5.** Concentrations of digestible energy (DE), metabolizable (ME) energy, and energy ratios in corn, fish waste (FW), supermarket waste (SMW), and fruits and vegetable waste (FVW) determined in experiment 1 (DM^1^ basis)

Item	Corn	FW	SMW	FVW	SEM	*P* value
DE, kcal/kg	3,928^b^	5,057^a^	5,071^a^	2,570^c^	98.91	<0.01
ME, kcal/kg	3,875^b^	4,820^a^	4,922^a^	2,460^c^	87.96	<0.01
Energy ratios						
DE:GE^2^	0.86^a^	0.79^a^	0.80^a^	0.62^b^	0.02	<0.01
ME:GE	0.85^a^	0.78^b^	0.76^b^	0.60^c^	0.02	<0.01
ME:DE	0.98^a^	0.95^c^	0.97^b^	0.96^bc^	0.004	<0.01

^a,b,c^Means with different superscripts within a row differ (*P <* 0.05).

^1^DM = dry matter.

^2^GE = gross energy.

The DE to GE ratios for FW (0.79), SMW (0.80), were not different from corn (0.86), but were greater (*P* < 0.05) than FVW (0.62). Corn had the greatest (*P <* 0.01) ME:GE (0.85) compared with FW (0.78), SMW (0.76), and FVW (0.60), which suggests that a greater proportion of the relatively high GE content in FW and SMW is not utilized by pigs compared with that of corn. This is expected because a large proportion of GE in FW and SMW comes from CP, which is less efficiently utilized as an energy source compared with lipids and starch. The DE to GE (0.62) and ME to GE (0.60) ratios of FVW were comparable to wheat bran (DE:GE = 0.60; ME:GE = 0.58) and greater than soybean hulls (DE:GE = 0.48; ME:GE = 0.46) as reported in NRC (2012), which suggests that FVW could be used as a low-energy, high-fiber ingredient in commercial swine diets, especially for gestating sows. Although SMW had a greater (*P* < 0.01) ME:DE than FW, there were no differences (*P* > 0.05) in ME:DE between SMW and FVW, or FVW and FW. Likewise, the DE:GE and ME:GE for FW and SMW were greater (*P <* 0.01) than FVW. However, the greater (*P <* 0.01) ME:DE in SMW than in FW was related to greater urinary GE loss from nitrogen (NRC, 2012). Excreted urinary energy was about 63.7 kcal/L (data not shown) greater in pigs fed FW group compared with those fed SMW, and the ratio of DE to ME has been shown to be affected by the CP content of the feedstuff (Morgan et al., 1975). High-protein intake can lead to greater excretion of urinary nitrogen resulting from increased catabolic activities, and urinary energy content is mainly related to the amount of nitrogen in urine (Morgan et al., 1975; Velayudhan et al., 2015). Increased urinary and fecal N excretion is highly related to excess dietary nitrogen intake, which often results in a lower percentage of N retention especially in diets with an AA imbalance (Noblet and Perez, 1993; Kerr and Easter, 1995). Thus, because the FW contained much greater nitrogen supply than in SMW (62.49% vs. 29.42%, respectively), N excretion in urine from the pigs fed the FW would be expected to be greater than for pigs fed SMW, resulting in the lower DE:ME. This is supported by the results from the AA digestibility experiment, where the sum of indigestible essential AA content was 2.6 g/kg in FW compared with 2.0 g/kg in SMW.

### In Vivo Phosphorus Digestibility

Phosphorus is the third most expensive component in swine diets and is an essential mineral because its role in many physiological functions, especially bone growth and mineralization (Cromwell, 2005). Total phosphorus content in FW was greater (*P <* 0.05) than in SMW and FVW (Table 6). However, the ATTD of P was greater (*P <* 0.05) in SMW than in FW and FVW. After adjustment for basal endogenous losses, STTD of P of SMW and FVW were greater (*P <* 0.05) than in FW. The total P content in FW was similar to the NRC (2012) value for fish meal (2.95% and 3.13%, respectively), but the P in fish meal (NRC, 2012) appears to be more digestible (STTD = 82%) than FW (STTD = 59%). It is unclear why the P digestibility in FW was much less than the value reported for fish meal in NRC (2012).

**Table 6.** Concentration, apparent total tract digestibility (ATTD), and standardized total tract digestibility (STTD) of phosphorus in fish waste (FW), supermarket waste (SMW), and fruit and vegetable waste (FVW) determined in experiment 2 (as-fed basis)

Item	FW	SMW	FVW	SEM	*P* value
Total P, %	2.95	0.38	0.26	—	—
ATTD P, %	56.00^b^	67.97^a^	52.95^b^	2.38	<0.01
STTD P, %	59.10^b^	81.94^a^	74.06^a^	2.38	<0.01
Standardized total tract digestible P, %	1.74^a^	0.31^b^	0.19^b^	0.07	<0.01

^a,b,c^Means with different superscripts within a row differ (*P* < 0.05).

Values for STTD were calculated by correcting values for ATTD for basal endogenous P loss using 190 mg/kg dry matter (DM) intake (NRC, 2012). The daily basal endogenous P loss was calculated by multiplying daily DM intake by 190 mg/kg DM.

Although the total P content in SMW was less than expected, the STTD of P was somewhat greater than expected, which was likely due to a substantial contribution of digestible P from meat, deli, and dairy products. In fact, the STTD of P in SMW (82%) was comparable to STTD of P in meat and bone meal (70%), meat meal (86%), and dried skim milk (98%) reported in NRC (2012).

In contrast, it was expected that the total P content in FVW would be relatively low (0.26%), but it was surprising that the STTD of P was very high (74%) because plant-derived foods and feed ingredients are known to contain high concentrations of phytic acid, which is an indigestible storage form of P in cereal grains and oil seeds. Phytic acid is poorly utilized by pigs due to the lack of phytase secreted in the gastrointestinal tract, which is the enzyme responsible for releasing phosphate groups from the phytate molecule (Reddy et al., 1982; Cromwell et al., 1995). Therefore, the STTD of P (NRC, 2012) in common feed ingredients such as corn (34%), soybean meal (48%), wheat (56%), sugar beet pulp (63%), and corn dried distillers grains with solubles (65%) is less than observed for FVW in this study. A plausible explanation for the high digestibility of P in FVW is unclear, but may be due to less P is being bound to phytate in fruits and vegetables compared with grains and grain-based ingredients. These results suggest that the FW source evaluated in this study is a concentrated source of P with relatively high digestibility, and although the total P content in SMW and FVW is relatively low, much of the P is digestible in pigs.

### In Vivo AA Digestibility

Thermal processing methods used during the dehydration of food waste may affect the digestibility and bioavailability of AA in food waste and must be considered when evaluating their use as digestible AA sources in swine diets (Qin et al., 1996; Anandharamakrishnan et al., 2007; Stein and Bohlke, 2007). The AID and SID of AA and CP were not different between FW and SMW (Table 7), but these sources contained greater (*P <* 0.05) AID and SID of all AA and CP than FVW. In fact, negative AID values were observed for most AA in FVW, and after accounting for basal endogenous loses of AA, negative values were still observed for SID of His, Cys, Gly, Pro, and Tyr in FVW. Digestibility of AA is reduced by increased concentrations of ADF and NDF in feed ingredients because fiber increases the secretion and reabsorption of endogenous AA, which affects the SID of AA (Lenis et al., 1996; Souffrant, 2001; Myrie et al., 2008). The SID of Pro and Gly exceeded 100% for FW, and Gly exceeded 100% for SMW. This may be explained by the potential biosynthesis of these dispensable AA from other AA in the enterocytes to produce mucin, which contributes to an increase in endogenous Gly and Pro losses when compared with other AA (Holmes et al., 1974; Reis de Souza et al., 2013). Other studies have reported similar losses of AA when high-fiber ingredients were fed to pigs to those observed in this study (Almeida et al., 2011; Reis de Souza et al., 2013; Oliveira and Stein, 2016).

**Table 7.** Apparent ileal digestibility (AID) and standardized ileal digestibility (SID) coefficients of crude protein (CP) and amino acids (AA) in fish waste (FW), supermarket waste (SMW), and fruits and vegetable waste (FVW) determined in experiment 3

	AID					SID				
Item, %	FW	SMW	FVW	Pooled SEM	*P* value	FW	SMW	FVW	Pooled SEM	*P* value
CP	83.1^a^	63.5^a^	−44.5^b^	8.0	<0.01	95.1^a^	89.3^a^	11.4^b^	7.9	<0.01
Indispensable AA										
Arg	92.0^a^	75.3^a^	−53.1^b^	13.6	<0.01	99.9^a^	96.0^a^	15.0^b^	13.6	<0.01
His	89.5^a^	76.3^a^	−64.0^b^	7.3	<0.01	95.2^a^	87.0^a^	−15.9^b^	7.3	<0.01
Ile	87.1^a^	78.4^a^	−34.1^b^	5.4	<0.01	94.0^a^	91.3^a^	8.1^b^	5.6	<0.01
Leu	88.2^a^	80.6^a^	−14.7^b^	5.1	<0.01	94.8^a^	92.7^a^	21.6^b^	5.2	<0.01
Lys	89.7^a^	77.9^a^	−35.5^b^	5.6	<0.01	94.7^a^	89.7^a^	9.9^b^	5.6	<0.01
Met	92.4^a^	82.9^a^	−7.4^b^	2.7	<0.01	95.0^a^	91.0^a^	24.4^b^	2.7	<0.01
Phe	87.7^a^	78.1^a^	−9.8^b^	5.4	<0.01	94.4^a^	92.3^a^	25.4^b^	5.4	<0.01
Thr	83.4^a^	68.2^a^	−64.2^ b^	9.3	<0.01	93.3^a^	91.4^a^	5.2^b^	9.3	<0.01
Trp	91.2^a^	83.2^a^	15.0^b^	5.9	<0.01	99.2^a^	96.1^a^	49.0^b^	5.9	<0.01
Val	83.2^a^	71.2^a^	−49.8^b^	6.8	<0.01	92.8^a^	89.9^a^	2.7^b^	6.8	<0.01
Dispensable AA										
Ala	88.2^a^	71.6^a^	−44.3^b^	7.2	<0.01	95.1^a^	90.7^a^	9.6^b^	7.2	<0.01
Asp	83.8^a^	71.1^a^	−5.6^b^	4.0	<0.01	90.8^a^	86.9^a^	27.2^b^	4.0	<0.01
Cys	65.8^a^	53.6^a^	−87.4^b^	10.1	<0.01	85.9^a^	82.6^a^	−20.2^b^	10.1	<0.01
Glu	88.3^a^	81.7^a^	5.7^b^	3.7	<0.01	94.3^a^	91.3^a^	35.8^b^	3.7	<0.01
Gly	89.1^a^	45.3^a^	−226. 0^b^	29.2	<0.01	102.9^a^	101.2^a^	−43.0^b^	29.2	<0.01
Pro	75.1^a^	−28.9^a^	−413.1^b^	88.2	<0.01	126.7^a^	96 .4^a^	−104.1^b^	88.3	<0.01
Ser	83.4^a^	68.4^a^	−39.8^b^	7.4	<0.01	92.8^a^	89.1^a^	13.3^b^	7.3	<0.01
Tyr	86.1^a^	73.9^a^	−91.1^b^	9.6	<0.01	93.7^a^	90.3^a^	−21.7^b^	9.6	<0.01
Total	86.4^a^	67.6^a^	−53.7^b^	9.6	<0.01	97.1^a^	91.4^a^	10.6^b^	9.6	<0.01

^a,b^Means with different superscripts within a row differ (*P* < 0.05).

The AID (89.7%) and SID (94.7%) of Lys in FW were greater than published AID (85%) and SID (86%) values for fish meal in NRC (2012). For SMW, the AID of Lys (77.9%) was less than that of soybean meal (86%; NRC, 2012), but the SID of Lys (89.7%) was similar to that of soybean meal (90%; NRC, 2012). The AID and SID of Met (92.4% and 95.0%, respectively) and Trp (91.2% and 99.2%, respectively) in FW were also greater than in fish meal (Met = 86.0% and 87.0%, respectively; Trp = 73% and 76%, respectively) reported by NRC (2012). Both AID and SID of Met and Trp in SMW (Met = 82.9% and 91.0%, respectively; Trp = 83.2% and 96.1%, respectively) were also greater than in soybean meal (Met = 80.0% and 85.0%, respectively; Trp = 87.0% and 89.0%, respectively) in NRC (2012). Considering the high concentration of Lys (4.12%), Met (1.57%), and Trp (0.62%) in FW compared with NRC (2012) values for fish meal, and the high SID of these AA in FW, it is an attractive substitute to traditional fish meal in swine diets.

### Comparison of In Vivo and In Vitro Digestibility of DM and Energy

**Figure 1.**
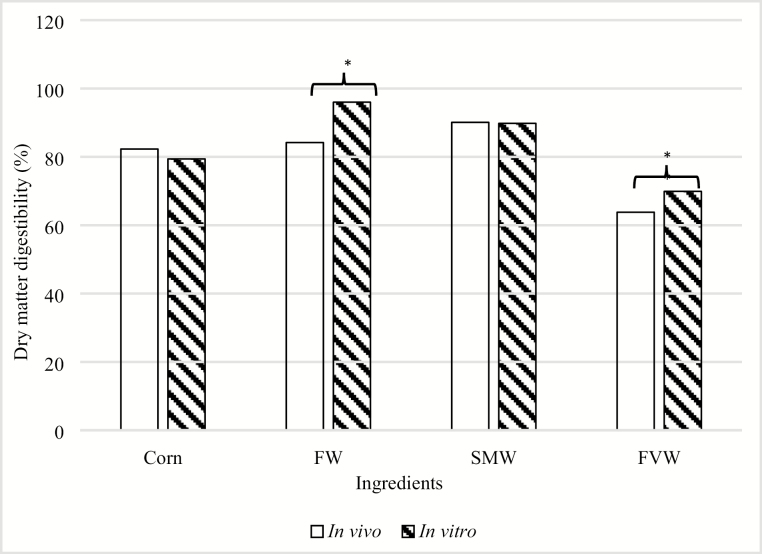
Comparison of in vivo vs. in vitro dry matter digestibility of corn (82.3% vs. 79.4%), fish waste (FW; 84.2% vs. 96.0%), supermarket waste (SMW; 90.1% vs. 89.8%), and fruit and vegetables waste (FVW; 63.8% vs. 69.9%). *Significant differences between in vitro and in vivo values (*P* < 0.05).

**Figure 2.**
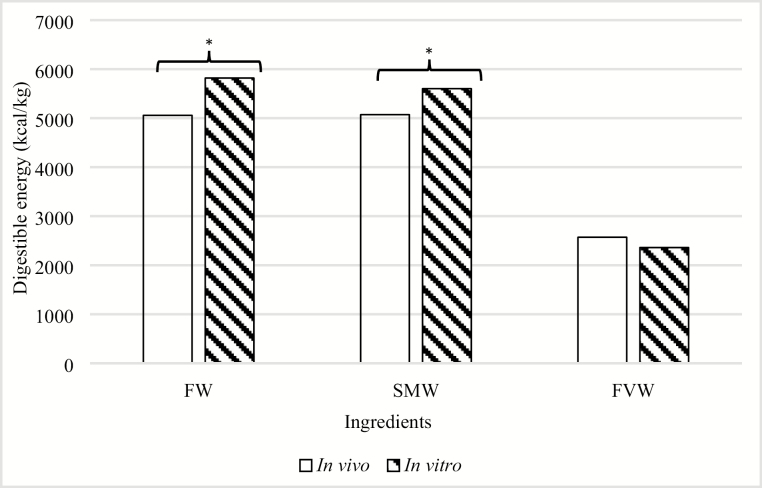
Digestible energy content determined using in vivo and in vitro methods (Noblet and Jaguelin-Peyraud, 2007) in fish waste (FW; 5,057 vs. 5,818 kcal/kg dry matter [DM]), supermarket waste (SMW; 5,071 vs. 5,602 kcal/kg DM), and fruits and vegetable waste (FVW; 2,570 vs. 2,360 kcal/kg DM). *Significant differences between in vitro and in vivo models (*P* < 0.05).

There is increasing interest for using rapid, accurate, low-cost alternative in vitro methods to evaluate the digestibility of feed ingredients instead of using animals in in vivo experiments to determine energy and nutrient digestibility (Święch, 2017). Therefore, the applicability of using a well-established in vitro assay to evaluate the energy and DM digestibility of three sources of food waste was evaluated in this study. In vitro digestibility of DM in corn, FW, SMW, and FVW was compared with the in vivo DM digestibility data obtained in experiment 1 (Figure 1). In vivo and in vitro digestibility of DM did not differ in corn (82.3% vs. 79.4%, respectively) or in SMW (90.1% vs. 89.8%, respectively), whereas differences were observed in FW (84.2% vs. 96.0%, respectively; *P* < 0.05) and FVW (63.8% vs. 69.9%, respectively; *P* < 0.05). When comparing the accuracy of using the in vitro method to estimate DM and nutrient digestibility, it is important to note that this method estimates the true DM digestibility of a feedstuff compared with in vivo determination, which includes endogenous losses in the determination of apparent DM digestibility (Reynolds, 2000; Kil et al., 2013). Therefore, our in vitro determined DE concentrations of FW and SMW were greater than the in vivo DE content (FW = 5,818 vs. 5,057 kcal/kg DM, respectively; SMW = 5,602 vs. 5,071 kcal/kg DM, respectively; *P* < 0.05; Figure 2). The differences in DE values obtained in the two methods may be explained by the endogenous losses of energy that occur using the in vivo method, whereas the in vitro method does not account for these endogenous losses (Reynolds, 2000; Kil et al., 2013). In contrast, there were no differences between in vitro and in vivo determined DE for FVW (2,360 vs. 2,570 kcal/kg DM; *P* > 0.05). These results suggest that the use of in vitro assays can accurately estimate DM digestibility in SMW, but overestimate DM digestibility in FW and FVW. Furthermore, in vitro determination of DE appears to be relatively accurate for FVW, but is overestimated for FW and SMW compared with in vivo determined values.

### Applicability of Using Prediction Equations to Estimate DE and ME in FW, SMW, and FVW

When equations for predicting DE and ME content based on chemical composition of food waste sources were evaluated, the equations from Noblet and Perez (1993) more closely predicted the in vivo determined DE of FW, SMW, and FVW than equations from Kerr et al. (2017; Tables 8 and 9). Within the Noblet and Perez’s (1993) equations used for predicting DE values, equation 1 most closely predicted the DE content of FW (observed = 5,057 kcal/kg DM vs. predicted = 5,234 kcal/kg DM), and equation 3 most closely predicted the DE content of SMW (observed = 5,071 kcal/kg DM vs. predicted = 4,909 kcal/kg DM). However, all three DE prediction equations from Noblet and Perez (1993) reasonably predicted DE content of FVW (observed = 2,570 kcal/kg DM vs. predicted = 2,731 kcal/kg DM; 2,736 kcal/kg DM; and 2,786 kcal/kg DM for equations 1, 2, and 3, respectively).

**Table 8.** Comparison of digestible energy (DE) content determined in vivo vs. predicted using equations from Noblet and Perez (1993) and Kerr et al. (2017) in fish waste (FW), supermarket waste (SMW), and fruits and vegetables waste (FVW)

Item	FW	SMW	FVW
Observed mean (in vivo)	5,057	5,071	2,570
Margin of error	259	131	251
Upper limit	5,316	5,202	2,822
Lower limit	4,798	4,940	2,319
Noblet and Perez—DE (1)	5,234	4,796	2,731
Noblet and Perez—DE (2)	4,517	4,831	2,736
Noblet and Perez—DE (3)	4,605	4,909	2,786
Kerr et al.—DE (8)	5,566	5,490	2,727
Kerr et al.—DE (9)	4,017	3,502	−66
Kerr et al.—DE (10)	3,991	5,053	4,931
Kerr et al.—DE (11)	4,104	3,961	1,019

**Table 9.** Comparison of metabolizable energy (ME) content determined in vivo vs. predicted using equations from Noblet and Perez (1993) and Kerr et al. (2017) in fish waste (FW), supermarket waste (SMW), and fruits and vegetables waste (FVW)

Item	FW	SMW	FVW
Observed mean (in vivo)	4,820	4,922	2,460
Margin of error	231	101	229
Upper limit	5,051	5,023	2,689
Lower limit	4,589	4,821	2,232
Noblet and Perez—ME (4)	4,090	4,759	2,749
Noblet and Perez—ME (5)	4,092	4,631	2,667
Noblet and Perez—ME (6)	4,810	4,596	2,662
Noblet and Perez—ME (7)	4,477	4,671	2,776
Kerr et al.—ME (12)	5,001	4,932	2,410
Kerr et al.—ME (13)	3,700	3,557	52
Kerr et al.—ME (14)	3,624	4,644	4,526
Kerr et al.—ME (15)	3,725	3,836	1,395

In contrast, when prediction equations from Kerr et al. (2017) were used to estimate the ME content of these food waste sources, they more closely estimated the in vivo ME values than the equations from Noblet and Perez (1993). Equation 12 from Kerr et al. (2017) most closely predicted the ME content for FW (observed = 4,820 kcal/kg DM vs. predicted = 5,001 kcal/kg DM), SMW (observed = 4,922 kcal/kg DM vs. predicted = 4,932 kcal/kg DM), and FVW (observed 2,460 kcal/kg DM vs. predicted 2,410 kcal/kg DM). Equation 12 from Kerr et al. (2017) also required use of the fewest input variables among the equations evaluated, and required only GE content to predict ME content in all three food waste sources. From these comparisons, it appears that equations from Noblet and Perez (1993) can be used to reasonably predict the DE content, whereas equations from Kerr et al. (2017) can be used to reasonably predict the ME values of these food waste sources.

We also evaluated the accuracy of using in vitro OM digestibility data in equations from derived by Noblet and Jaguelin-Peyraud (2007), and results are shown in Table 10. Most of these equations closely predicted the DE content of FW, SMW, and FVW relative to the in vivo determined values. For instance, equations 17, 18, and 19 from Noblet and Jaguelin-Peyraud (2007) reasonably predict the DE content of FW (observed = 5,057 kcal/kg DM vs. predicted = 4,948, 4,885, and 4,852 kcal/kg DM, respectively), and equation 18 reasonably predicted the DE content of SMW (observed = 5,071 kcal/kg DM vs. predicted = 4,978 kcal/kg DM). Last, equations 18 and 19 were relatively accurate in predicting the DE content of FVW (observed = 2,570 kcal/kg DM vs. predicted = 2,814 and 2,696 kcal/kg DM, respectively).

**Table 10.** Prediction of digestible energy (DE) content in fish waste (FW), supermarket waste (SMW), and fruits and vegetables waste (FVW) using a combination of in vitro organic matter digestibility and nutrient content using equations from Noblet and Jaguelin-Peyraud (2007)

Item	FW	SMW	FVW
Observed value (in vivo)	5,057	5,071	2,570
Margin of error	259	131	251
Upper limit	5,316	5,202	2,822
Lower limit	4,798	4,940	2,319
Noblet and Jaguelin-Peyraud—DE (16)	4,333	4,052	3,155
Noblet and Jaguelin-Peyraud—DE (17)	4,948	5,410	3,135
Noblet and Jaguelin-Peyraud—DE (18)	4,885	4,978	2,814
Noblet and Jaguelin-Peyraud—DE (19)	4,852	4,750	2,696

These results suggest that using selected published prediction equations, DE and ME content of these three food waste sources can be reasonably estimated and be comparable to values obtained from in vivo experiments. However, the accuracy of DE and ME prediction equations varies among sources of food waste based on their nutritional characteristics. It appears that using in vitro OM digestibility data in the Noblet and Jaguelin-Peyraud’s (2007) equations resulted in the greatest accuracy of predicted DE for all the sources of food waste.

In conclusion, results from the present study indicate that both FW and SMW are excellent sources of DE, ME, and digestible AA for pigs and could be used to partially replace corn and soybean meal in swine diets to reduce environmental impact. Specific prediction equations from Noblet and Perez (1993) and Kerr et al. (2017) can be used to provide reasonable estimates of DE or ME content, respectively, of food waste sources. Furthermore, the use of in vitro digestibility methods to determine digestible OM content of food waste sources, along with DE prediction equations from Noblet and Jaguelin-Peyraud (2007), can be used to reasonably estimate the DE content of FW, SMW, and FVW of these food waste sources.
